# Quantitative MRI and Clinical Assessment of Muscle Function in Adults With Cerebral Palsy

**DOI:** 10.3389/fneur.2021.771375

**Published:** 2021-11-10

**Authors:** Christian Svane, Christian Riis Forman, Aqella Rasul, Christian Hammer Nielsen, Jens Bo Nielsen, Jakob Lorentzen

**Affiliations:** ^1^Department of Neuroscience, University of Copenhagen, Copenhagen, Denmark; ^2^Elsass Foundation, Charlottenlund, Denmark; ^3^Department of Diagnostic Radiology, Rigshospitalet, Copenhagen University Hospital, Copenhagen, Denmark

**Keywords:** magnetic resonance imaging (MRI), cerebral palsy, muscle composition, muscle size, fat fraction, countermovement jump, maximal voluntary contraction

## Abstract

**Aim:** To relate quantitative magnetic resonance imaging (MRI) of ankle plantar flexor muscles to clinical functional tests in adults with cerebral palsy (CP) and neurologically intact (NI) adults.

**Methods:** Eleven adults with CP (aged 41 ± 12, GMFCS level I-II) and 11 NI adults (aged 35 ± 10) participated in this case-control study. We used MRI to assess muscle volume and composition of the triceps surae muscles. We quantified muscle function as maximal voluntary plantarflexion (MVC) torque and countermovement jump (CMJ) height.

**Results:** Compared to NI adults, the MRI intramuscular fat fraction estimate was significantly higher and MRI muscle volume and functional abilities (MVC and CMJ) significantly lower in adults with CP. In NI adults, but not adults with CP, MRI muscle volume correlated significantly with MVC and CMJ. In adults with CP, the estimate of intramuscular fat levels correlated significantly with jump height in a CMJ.

**Discussion:** This study shows reduced muscle volume and altered muscle composition in adults with CP. Muscle composition appears to provide a better marker than muscle volume of reduced muscle function and impaired performance in this population. Measurements of muscle composition could be used in the assessment of neuromuscular impairments and in the determination of rehabilitation protocols in individuals with neurological disorders.

## Introduction

Cerebral palsy (CP) is a disorder caused by a non-progressive disturbance of the developing brain occurring before or at the time of birth (1). Although the disorder is non-progressive, adults with CP show declining functional abilities at an early age resulting from, and further contributing to, increased sedentary lifestyle, deterioration of functional abilities and increased risk of lifestyle related diseases (2). As a result, these impairments persist and are often aggravated in adulthood (3–5). In many adults with CP, the disorder is associated with functional impairments such as muscle weakness, muscle contractures and reduced muscle function (6, 7). Although CP is primarily a neurological disorder, a large part of this functional decline may be related to secondary muscular adaptations (2). Clinically, these secondary adaptations can be assessed using quantitative magnetic resonance imaging (MRI) of muscle volume and composition.

Muscle volume can be derived from MRI scans by drawing the region of interest (ROI) on obtained image slices of the muscle in question and calculating the total volume of the ROI. In neurologically intact (NI) children and adults, it is well-documented that muscle function (often quantified as muscle strength) is closely related to muscle size, e.g., measured using MRI (8). In the CP population however, muscle size and muscle function does not seem to be as closely related—at least not in children (aged 6–10 years) (9). Although the lack in muscle size-function relationship initially seem counter-intuitive, this might be—at least partly—explained by the sedentary nature of the CP population (10). The sedentary nature of the CP population may cause large accumulations of intramuscular fat content in some individuals, thereby reducing the amount of contractile tissue per unit of muscle volume (11). Furthermore, inflammatory cytokines secreted by the intramuscular fat might cause further reductions in contractile force (12), again disturbing the relationship between muscle size and function. In addition to the accumulation of intramuscular fat content, the development of muscle contractures are also prevalent in the CP population (13). As muscle contractures are related to an accumulation of intramuscular connective tissue (14), the presence of contractures will also reduce the amount of contractile tissue per unit of muscle volume and therefore impact the relationship between muscle size and function. Increased stiffness and reduced joint mobility, characteristic of muscle contractures, have indeed been shown to be negatively correlated with parameters of gait function in adults with CP (15).

In other populations with muscle impairments [e.g., sarcopenia and muscular dystrophies (16, 17)], the importance of using measures of muscle composition in addition to muscle size as a determinant of muscle function has already been shown (18). One way of quantifying muscle composition is by creating separate water and fat images based on a 2-point echo gradient Dixon MRI sequence (19, 20). Based on the images showing primarily water or fat, respectively, it is possible to calculate what is generally described as the intramuscular fat fraction. This approach of quantifying muscle composition has been used in past studies on both children (21) and adults (11) with CP. Both studies report increases in this MRI measure of muscle composition and argue that this reflects increased levels of intramuscular fat in the CP population. Here, it is however important to note that enlarged amounts of other intramuscular tissue types, such as the enlarged connective tissue levels found in individuals with muscle contractures, will affect this measure to some extent. We have nevertheless continued to use the term “fat fraction” to denote this MRI measure throughout the present paper in accordance with the literature.

In this study, we aimed to investigate the relationship between MRI measures of muscle volume and composition and clinical measures of muscle function in adults with CP compared to NI adults.

## Materials and Methods

We designed this study as a case-control study. All experiments were performed at the University of Copenhagen between March and August 2020 and participants were enrolled on an ongoing basis. The study was approved by the ethics committee for the capital region of Denmark (H-17039575) and all study procedures were performed in accordance with the Helsinki declaration. Prior to the experiments, informed consent was obtained from all participants.

### Participants

We recruited 11 adults with CP {aged 41 ± 12; seven female, GMFCS [Gross Motor Function Classification System (22)] level I-II} and 11 NI adults (aged 35 ± 10; five female) between March and June 2020. Ten NI adults were right leg dominant; one was left leg dominant. Adults with CP were recruited through a posting on the Elsass Foundation website. Eligibility criteria for adults with CP were: 18–60 years of age and GMFCS level I-II. Participants are described in detail in Table 1. Neurological examination was performed by an experienced neurological physiotherapist (JL). Four of the participants (13, 15, 20, and 22) were considered diplegic clinically, but all participants showed a difference between the two sides in either muscle force, Modified Ashworth Scale (MAS) or Range of Movement (ROM) in the ankle joint. It was therefore decided to identify a most affected (MA) and least affected (LA) leg in all subjects. No participants had received lower body Botulinum Toxin treatment in the past 12 months prior to the examinations. Although some subjects had a history of lower body tendon-lengthening surgery, no subjects had lower body tendon-lengthening surgery performed in the past 12 months prior to the examinations.

**Table 1:** Subject characteristics.

													**Strength**
**ID**	**CP/**	**Sex**	**Weight**	**Height**	**BMI**	**Age**	**GMFCS**	**Surgery**	**MAS pf**	**Reflex**	**ROM**	**Most**	**L ankle**	**L knee**	**R ankle**	**R knee**
	**NI**	**(M/F)**	**(kg)**	**(cm)**	**(kg/m** ^**2**^ **)**	**(years)**		**(yes/no)**	**pf**	**achilles**	**ankle**	**affected**	**(pf/df)**	**(ext/flex)**	**(pf/df)**	**(ext/flex)**
									**(L/R)**	**(L/R)**	**(L/R)**	**side**				
10	CP	M	71	172	24	54	II	No	1/0	-/-	−10°/Full	Left	4/2	4/3	4/3	4/3
11	CP	M	74	185	22	48	II	No	2/0	+/Normal	−10°/Full	Left	3/2	3/2	5/5	5/5
12	CP	F	84	174	28	60	II	Yes	2/0	-/Normal	−20°/Full	Left	3/3	4/4	5/5	5/5
13	CP	F	55	165	20	28	I	No	2/2	+/+	−10°/−10°	Left	4/3	5/5	4/3	5/5
14	CP	F	110	176	36	35	I	Yes	0/2	Normal/-	Full/−15°	Right	5/5	5/5	3/0	3/3
15	CP	F	74	169	26	35	I	No	3/3	+/+	−30°/−25°	Left	5/4	5/4	5/4	5/4
16	CP	F	62	160	24	43	I	No	1+/0	-/Normal	−10°/Full	Left	4/4	4/4	5/5	5/5
17	CP	F	46	161	18	34	I	No	1+/1	+/Normal	Full/Full	Left	4/4	4/4	4/4	4/4
19	CP	M	69	183	21	20	I	Yes	?/2	+/+	Full/−25°	Right	5/5	5/5	4/3	4/4
20	CP	F	93	168	33	60	II	Yes	1/2	-/Normal	−5°/Full	Left	4/3	4/3	5/4	4/4
22	CP	M	72	175	24	35	I	Yes	2/3	+/-	−10°/−20°	Right	4/3	4/3	4/3	3/3
1	NI	M	70	173	23	57										
2	NI	M	95	190	26	47										
3	NI	M	90	190	25	28										
4	NI	F	55	162	21	22										
5	NI	M	84	173	28	27										
7	NI	M	68	181	21	34										
8	NI	M	83	190	23	30										
9	NI	F	60	175	20	25										
18	NI	F	58	167	21	45										
21	NI	F	59	167	21	38										
23	NI	F	67	158	27	37										

*Detailed subject characteristics of all participants in the study. Reflexes are given as “-” if reduced or absent and as “+” if exaggerated. Achilles tendon lengthening surgery: ID12 (left side), ID14 (right side), ID19 (right side), ID20 (left side), ID22 (right side). ID14 had tendon lengthening surgeries performed in the left and right knee joint. pf, plantarflexion; df, dorsiflexion; ext, extension; flex, flexion. Due to an ankle injury, we were not able to evaluate the MAS of the left ankle in participant 19*.

### Experimental Protocol

**Figure 1:**
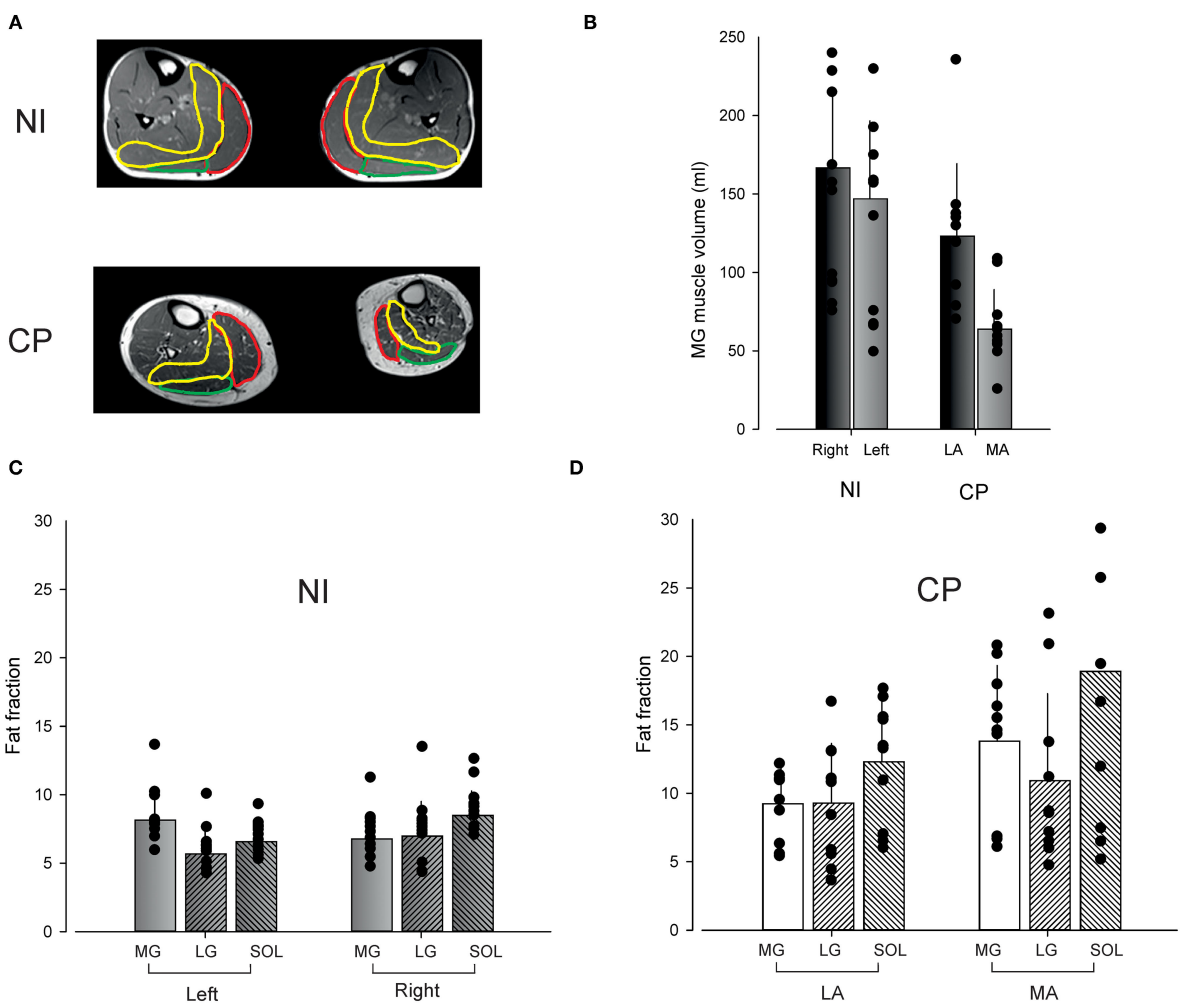
Muscle volume and composition. Muscle volume and intramuscular fat fraction in neurologically intact adults (NI) and adults with Cerebral Palsy (CP) measured by MR. **(A)** Shows examples of scans from an NI individual and an individual with CP. The medial gastrocnemius muscle has been marked in red, the lateral gastrocnemius muscle in green and the soleus muscle in yellow. **(B)** Shows the medial gastrocnemius muscle volume on the most affected side in adults with CP and on the right side in NI adults. **(C)** Shows the calculated fat fraction for each of the three triceps surae muscles on the left and right side in NI adults. **(D)** Shows the fat fraction for adults with CP for their least affected (LA) and most affected (MA) leg. Bars show the average fat fraction with vertical lines depicting standard deviations. Data from individual subjects have been superimposed as black circles on top of each of the bars.

**Figure 2:**
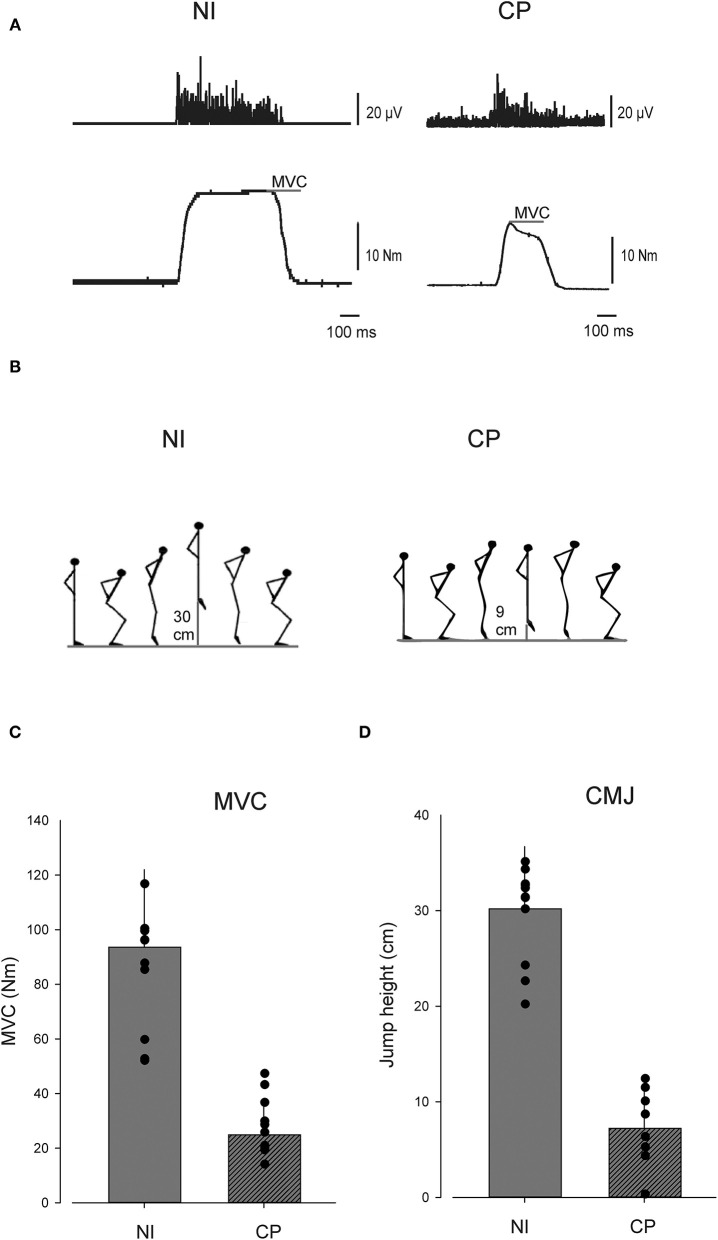
Muscle function. **(A)** Show EMG and torque data from single individuals performing plantar flexion maximal voluntary contraction (MVC), **(B)** depicts a countermovement jump (CMJ). **(C)** Depicts MVC and **(D)** depicts CMJ in the cerebral palsy (CP) group and in the neurologically intact (NI) group. In **(C,D)** individual values are depicted as dots and group means as bars. Vertical lines indicate SD.

We used quantitative MRI to assess the muscle volume and muscle composition of the triceps surae muscles of the left and right leg (depicted in Figure 1). We assessed plantar flexor muscle function by measuring the torque eliciting a maximal voluntary contraction (MVC) and the jump height during a countermovement jump (CMJ) (depicted in Figure 2).

### Neurological Examination

All individuals with CP were neurologically examined by an experienced neurological physiotherapist (J.L.). Here, MAS, reflexes, ROM, and strength of the lower body were evaluated. The ROM of the ankle joint was evaluated by slowly moving the joint through the movement range, noting positions of maximal plantarflexion and dorsiflexion. These positions were reached without causing discomfort to the participants. The MAS score of the plantarflexors was then determined and the presence and possible exaggeration of the Achilles reflex evaluated using a reflex hammer. Lastly, voluntary strength of the lower body was evaluated on a scale from 0 to 5 using the MRC scale (23).

### MRI

During the MRI measurements, the participant was placed in a supine feet first position on the bed with the knees fully extended and the ankles in a plantarflexed position of ~110 degrees. Knees were resting on a pillow for comfort and the legs tied together at the ankles by a piece of cloth to prevent external rotation of the hip during scans. MRI images were acquired on a 3.0 Tesla Siemens Magnetom Verio system (Siemens Healthcare GmbH, Germany) using a 3T body matrix flex coil placed over the lower leg covering from knee until the ankle. Axial turbo spin echo T1-weighted images were acquired with TR/TE = 1,010/19.0 ms, 140° flip angle, 1.5 × 1.2 mm in-plane resolution, 6.0 mm slice thickness. A 2-point echo gradient Dixon sequence was performed with TR/TE1/TE2 = 5.59/2.45/3.675 ms, 9° flip angle, 2.1 × 1.4 mm in-plane resolution, 3.0 mm slice thickness. The total duration of the scan was ~6 min. Two localizers were performed lasting 53 and 51 s. T1-weighted scan lasted 2 min and 14 s while the Dixon sequence lasted 1 min and 42 s. Separate water and fat images were calculated online within the scanner software.

ROIs were drawn manually onto T1-weighted images around the medial gastrocnemius (MG) muscle for each image slice on both the left and right leg using 3D Slicer (www.slicer.org) (24) and total volumes calculated. We did not calculate the muscle volume of the lateral gastrocnemius (LG) muscle or the soleus muscle as we were not able to identify the entire ROI in the acquired scans. For the MG muscle, the ROIs used in the muscle volume calculation were then overlaid on the Dixon water and fat images and intensity values extracted. For the LG muscle and the soleus muscle, intensity values were extracted from an imaging slice from the bulk of the muscle. An estimate of the fraction of fat in the ROI was subsequently calculated using the following formula:


$$\begin{array}{l} Fat\;percentage=\frac{\text{Fat intensity}}{\text{Fat intensity }+\text{ Water intensity}} \end{array}$$

### MVC

When performing the isometric MVC measurements, the participant was seated comfortably in an armchair with the examined leg flexed in the hip to 120, the knee flexed to 110, and the ankle in 90 degrees plantar flexion. The foot was strapped to a footplate, which was connected to a dynamometer that could measure the torque exerted on the footplate. We chose an ankle joint position of 90 degrees to ensure both near-maximal muscle force-length relationship and that all subjects were able to perform the task. It is however worth noting that individual differences in muscle architecture (e.g., tendon lengths) could influence the force-generating abilities in this specific ankle joint position in CP (25). The device is described in detail in a previous study (26). The participants were verbally encouraged to perform a quick and strong plantar flexion without activating the muscles in the thigh. We performed three trials at intervals of 30 s and selected the one with the largest torque measured.

### CMJ

As a functional measure of plantar flexor muscle function, we used the jump height during a CMJ measured using the MyJump2 app. CMJ is widely used to assess the capability to produce rapid force during a dynamic movement (27). CMJ measured using the app has high validity and reliability both when used on NI adults and adults with CP (28–30). In a study on footballers with CP, the intraclass correlation coefficients were found to vary from 0.92 to 0.96 (*p* ≤ 0.001) (30). In NI adults, the CMJ measured using the app showed excellent agreement with results obtained using a force-plate (*r* > 0.99) and an almost perfect intraclass correlation coefficients (0.997) (29). In the CMJ, the participants started with their hands fixed at the waist. At the command of the evaluator, the participants performed a CMJ. The participants were instructed to jump as high as possible. After one practice jump, participants had three attempts with 1–2 min of rest in between. We reported the maximum jump height for each participant.

### Statistical Analysis

All values are given as mean ± SD. All statistical analyses were performed using RStudio (31). Normality was tested by visual inspection of histograms. We compared the normally distributed muscle volumes between groups by independent *t*-tests and correlated the anatomical variables (muscle volume and fat fraction) to the functional variables (MVC and CMJ) by performing Pearson's correlations to investigate any significant linear relationships (*p* < 0.05). Two-way ANOVA was used to test for differences in MG muscle volume in the two legs in NI adults and adults with CP (Factors: leg and group). To do the analysis, the left leg was designated MA and the right leg LA in NI adults. Three-way ANOVA was used to test for significant interaction between MRI intramuscular fat fraction in each of the triceps surae muscles in both legs of adults with CP and NI adults (Factors: muscle, leg, and group).

## Results

### Participant Characteristics

We found no significant differences in age (*p* = 0.28), weight (*p* = 0.78), height (*p* = 0.42), and BMI (*p* = 0.38) between the participants in the CP and NI group.

### Muscle Volume

MG muscle volume on the left and right side in NI adults and on the MA and LA side in adults with CP is shown in Figure 1B. In NI adults, MG muscle volume was 147 ± 50 ml on the left side and 167 ± 46 ml on the right side. In adults with CP, MG muscle volume was 63 ± 25 ml on the MA side and 123 ± 46 ml on the LA side.

Two-way ANOVA revealed no significant interaction between leg and group (*F* = 2.25; *p* = 0.14). Adults with CP were however found to have significantly smaller muscle volume in the two legs than NI adults (*F* = 22.6; *p* < 0.001) and the MG muscle volume of the LA/right leg was found to be significantly larger than the MA/left leg MG muscle volume (*F* = 8.8; *p* < 0.01). Subsequent *t*-tests showed that the muscle volume was significantly smaller on the MA side in adults with CP as compared to the LA side (*p* < 0.001) and to both the left (*p* = 0.03) and right side in NI adults (*p* < 0.001). The MG muscle volume on the left side was also significantly smaller than the right side in NI adults (*p* < 0.001).

### Muscle Composition

On average, the fat fraction estimate was 15.4 ± 7.3% in the most affected leg in the CP population as compared to 7.2 ± 1.1% in NI adults and 9.2 ± 2.2% on the least affected side (Figures 1C,D). In NI adults, the fat fraction on the left side was 8.1 ± 2.1% in the MG muscle, 5.7 ± 1.6% in the LG muscle, and 6.6 ± 1.2% in the soleus muscle. On the right side, the fat fraction was 6.8 ± 1.8% in the MG muscle, 7.0 ± 2.5% in the LG muscle, and 8.5 ± 1.8% in the soleus muscle. In adults with CP, the fat fraction on the LA side was 9.2 ± 2.6% in the MG muscle, 9.3 ± 4.4% in the LG muscle, and 12.3 ± 4.4% in the soleus muscle, whereas the fat fraction on the MA side was 13.8 ± 5.5% in the MG muscle, 10.9 ± 6.3% in the LG muscle, and 18.9 ± 11.4% in the soleus muscle.

Three-way ANOVA revealed a significant interaction between group and leg (*F* = 4.9; *p* = 0.0024), whereas no interaction with muscle was found suggesting that all three muscles were equally affected (*F* = 1.2; *p* = 0.3). *Post-hoc* tests revealed significantly larger fat fraction estimates in the three muscles on the most affected side in adults with CP as compared to the least affected side (*p* < 0.001) and NI adults (*p* < 0.001). Fat fraction estimates were also significantly larger on the least affected side in CP as compared to NI adults (*p* = 0.003). There was no significant difference in fat fraction between the two legs in NI adults (*p* = 0.59).

### Muscle Strength and Function

Adults with CP showed a significant reduction in MVC-elicited plantarflexion torque and CMJ jump height compared to NI adults (Figure 2). MVC was on average 0.26 ± 0.12 Nm in the CP group and 0.975 ± 0.28 Nm in the NI group (*p* < 0.001). CMJ jump height was on average 7.2 ± 4.3 cm in the CP group and 30.2 ± 6.2 cm in the NI group (*p* < 0.001).

### Correlations

**Figure 3:**
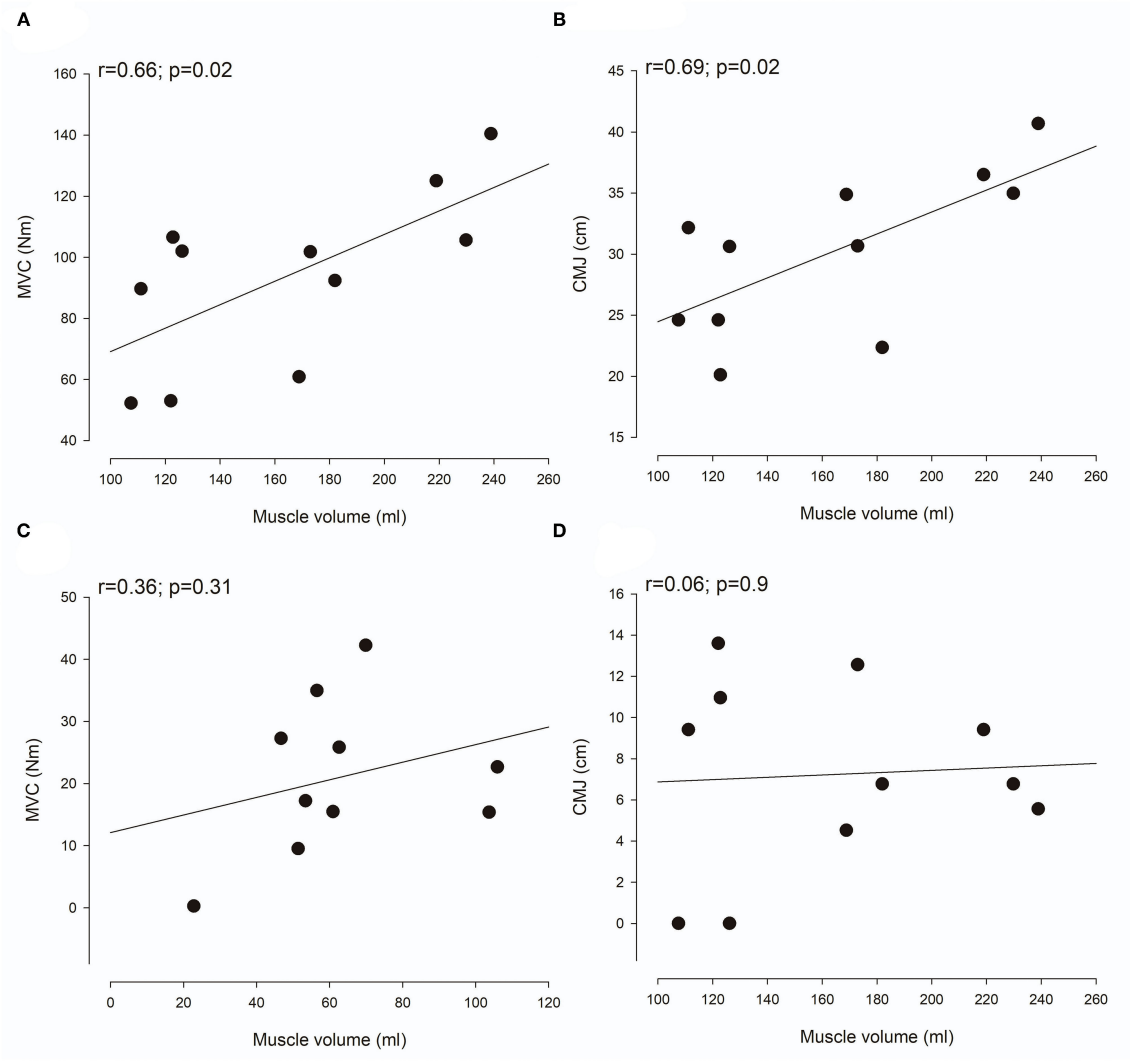
Relationship between muscle volume and function. **(A,C)** show the correlation between muscle volume of the medial gastrocnemius muscle and maximal voluntary contraction (MVC) in neurologically intact (NI) adults **(A)** and adults with Cerebral palsy (CP) **(C)**. **(B,D)** show the correlation between muscle volume of the medial gastrocnemius muscle and countermovement jump (CMJ) in NI adults **(B)** and adults with CP **(D)**. Each plot shows the trend line as well as individual data points.

MG Muscle volume was significantly correlated with MVC (Figure 3A; *r* = 0.66; *p* = 0.02) and CMJ (Figure 3B; *r* = 0.69; *p* = 0.02) in NI adults. No significant correlation was found between MG muscle volume and either MVC (Figure 3C; *r* = 0.36; *p* = 0.31) or CMJ (Figure 3D; *r* = 0.06; *p* = 0.9) in adults with CP.

**Figure 4:**
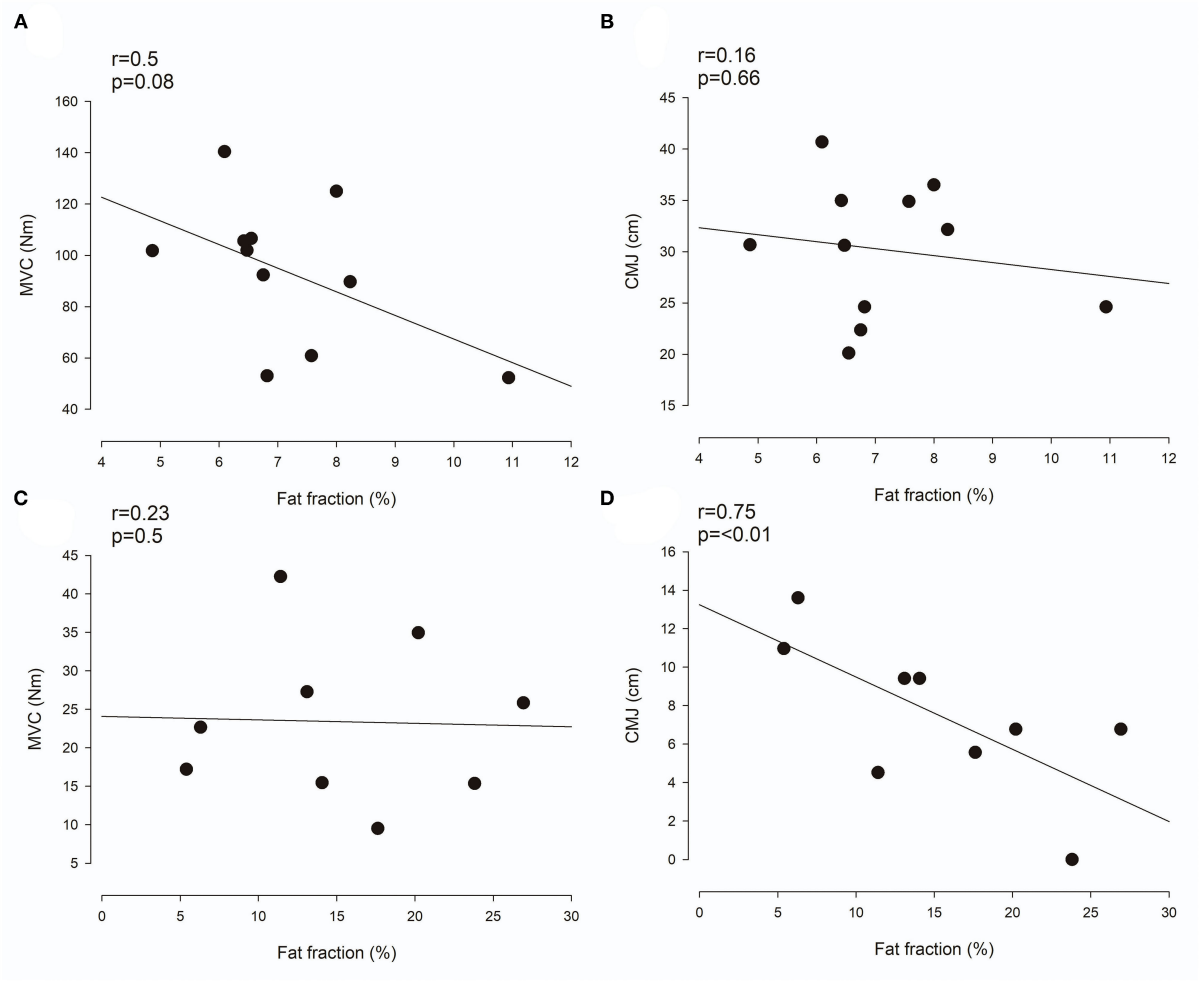
Relationship between muscle composition and function. **(A,C)** Shows the correlation between the mean fat fraction in the triceps surae muscles and maximal voluntary contraction (MVC) in neurologically intact (NI) adults **(A)** and adults with Cerebral Palsy (CP) **(C)**. **(B,D)** show the correlation between the mean fat fraction in the triceps surae muscles and countermovement jump (CMJ) in NI adults **(B)** and adults with CP **(D)**. Each plot shows the trend line as well as individual data points.

In NI adults, the fat fraction estimate of the three triceps surae muscles was not correlated with either MVC (Figure 4A; *r* = 0.5; *p* = 0.08) or CMJ (Figure 4B; *r* = 0.16; *p* = 0.66). For adults with CP, a highly significant correlation was in contrast found between the fat fraction estimate and CMJ (Figure 4D; *r* = 0.75; *p* < 0.01), whereas no correlation was found with MVC (Figure 4C; *r* = 0.23; *p* = 0.5).

### Relation to Clinical Parameters

Adults with CP classified by GMFCS level I showed lower fat fraction than adults classified by GMFCS level II (9.1 ± 4.1% and 17.5 ± 3.0%, respectively; *p* < 0.01). We found no difference in MG muscle volume between adults classified with GMFCS level I and adults classified by GMFCS level II (90 ± 34 ml and 98 ± 59 ml, respectively; *p* < 0.73). Adults classified by GMFCS level I jumped higher (9.4 ± 4.1 cm) than adults classified by GMFCS level II (6.5 ± 4.5 cm), but this was not a significant difference (*p* = 0.8). There was no relation between the other clinical parameters evaluated (Table 1) and either the estimated fat fraction or CMJ.

## Discussion

We found MRI MG muscle volume to be decreased and MRI intramuscular fat fraction levels to be increased in adults with CP compared to a NI control group. Thus, the present study provides further evidence of secondary adaptations in muscle volume and composition in adults with CP. A significant correlation between intramuscular fat fraction level and CMJ jump height could indicate that the muscle composition of triceps surae muscles is an important marker of impaired functional capacity in adults with CP. In contrast, MG muscle volume appears not to be a good marker of functional performance in adults with CP.

### Quantitative MRI as Marker of Functional Capacity in Adults With CP

Despite no differences in weight, height, BMI, or age, we found MRI muscle volume to be decreased, intramuscular fat fraction levels to be increased, and clinical measures of muscle function to be decreased in adults with CP compared to NI adults. This is in accordance with previous studies, investigating muscle volume (9, 32–34), composition (11, 21), and function (34–37) in both children and adults with CP.

In accordance with previous studies (8), we found a clear and significant relationship between muscle volume and clinical measures of muscle function in NI adults. However, similar to the findings of Reid et al. (9), the magnitude of the relationship between muscle size and clinical measures of muscle function was not as large in individuals with CP. We believe that this might be partly explained by large accumulations of intramuscular fat and muscle contracture-related connective tissue in some individuals with CP. Intramuscular accumulation of fat and connective tissue reduces the amount of contractile tissue per unit of muscle volume and thus disturbs the otherwise linear relationship between muscle size and function. In addition, the relationship between muscle size and function is also likely to be impacted by factors such as alterations in neural drive and increased co-contraction in some individuals with CP (9). It therefore seems as though other measures than muscle volume would constitute a better marker of muscle function in the CP population. In fact, we found a strong correlation between the intramuscular fat fraction, an MRI muscle composition measure thought to primarily reflect fat infiltration, and muscle function measured as jump height in a CMJ in adults with CP in this study (*r* = 0.75, *p* < 0.01). We find this to indicate that MRI measures of muscle composition might constitute such a marker in the CP population. Here, it should be added that other measures of muscle composition, e.g., ultrasound echo intensity (18), are likely to also constitute such markers in the CP population. In NI adults, muscle composition was not related to clinical measures of muscle function. We believe that this might be due to the relatively large homogeneity of the group, causing only little dispersion in muscle composition values (Figure 4). Despite the relatively large homogeneity of the group, the correlation between muscle composition and MVC did approach the level of significance (*r* = 0.5, *p* = 0.08). In terms of the correlation between muscle composition and CMJ jump height in NI adults however, we did not find convincing evidence (*r* = 0.16, *p* = 0.66).

As MRI is used in the clinic, the finding of MRI muscle composition as a potential marker of functional abilities could be of clinical importance, e.g., in in the assessment of neuromuscular impairments and in the determination of rehabilitation programs and goals.

### Limitations

Several important study limitations need to be considered. We designed this study as an observational case-control study and causative conclusions are therefore naturally not possible. We do however still believe that the study provides valuable information about the potential use of MRI markers of functional capacity in the CP population. Investigating the relationship between MRI measures of the plantar flexor muscles on the most affected side with a broad measure of lower body muscle function such as CMJ poses a limitation to the study. It is evident that many other factors, including familiarity, muscle coordination, the development of larger muscle groups, physical activity level, and the ROM of ankle, knee and hip joints will affect the jump height in a CMJ. Furthermore, although all adults with CP had a MA and a LA side, it is natural to believe that participants with hemiplegia can compensate more with the less affected side than participants with diplegia. Including a test of muscle function which primarily involved the MA side or primarily involved plantar flexor muscle function could therefore have been a valuable addition to this study. We measured CMJ using the smartphone app MyJump2. Although CMJ measured using MyJump2 has been reported to have high validity and reliability, it is subject to human error and not as thoroughly tested as CMJ measured using a force platform. All this aside, we believe that the strong correlations between muscle size and CMJ jump height in NI adults and between muscle composition and CMJ jump height in adults with CP confirms the validity of including this measure of muscle function.

## Conclusion

The present study shows that adults with CP have decreased MRI muscle size, changed MRI muscle composition, and decreased functional capacity compared to NI adults. MRI muscle composition appears to be closer related to functional performance than MRI muscle volume in adults with CP. We therefore suggest that muscle composition measured using MRI may be an important anatomical marker for reduced functional capacity in adults with CP. Measurements of muscle composition could be used in the assessment of neuromuscular impairments and in the determination of rehabilitation protocols in individuals with neurological disorders.

## Data Availability Statement

The raw data supporting the conclusions of this article will be made available by the authors, without undue reservation.

## Ethics Statement

The studies involving human participants were reviewed and approved by the Ethics Committee for the capital region of Denmark (H-17039575). The participants provided their written informed consent to participate in this study.

## Author Contributions

CS, CF, AR, and JL were involved in data acquisition. CS, CF, AR, JN, and JL were involved in analysis and interpretation of data. JN prepared figures. CS wrote the first draft of the manuscript. All authors substantially contributed to the conception and design of the study, revised the manuscript critically for important intellectual content, and approved the version of the manuscript to be published.

## Funding

This study was funded by a grant from the Elsass Foundation.

## Conflict of Interest

The authors declare that the research was conducted in the absence of any commercial or financial relationships that could be construed as a potential conflict of interest.

## Publisher's Note

All claims expressed in this article are solely those of the authors and do not necessarily represent those of their affiliated organizations, or those of the publisher, the editors and the reviewers. Any product that may be evaluated in this article, or claim that may be made by its manufacturer, is not guaranteed or endorsed by the publisher.

## Abbreviations

BMI: body mass index
CMJ: countermovement jump
CP: Cerebral Palsy
GMFCS: gross motor function classification scale
LA: least affected
MA: most affected
MAS: modified Ashworth scale
MG: medial gastrocnemius
MRI: magnetic resonance imaging
MVC: maximal voluntary contraction
NI: neurologically intact
ROI: region of interest
ROM: range of motion.
